# Biphasic Scaffolds from Marine Collagens for Regeneration of Osteochondral Defects

**DOI:** 10.3390/md16030091

**Published:** 2018-03-13

**Authors:** Anne Bernhardt, Birgit Paul, Michael Gelinsky

**Affiliations:** Centre for Translational Bone, Joint and Soft Tissue Research, University Hospital Carl Gustav Carus and Faculty of Medicine of Technische Universität Dresden, Fetscherstraße 74, 01307 Dresden, Germany; birgit.paul@tu-dresden.de (B.P.); michael.gelinsky@tu-dresden.de (M.G.)

**Keywords:** jellyfish collagen, mineralized salmon collagen, osteochondral tissue engineering, biphasic scaffold, osteochondral medium, alginate

## Abstract

Background: Collagens of marine origin are applied increasingly as alternatives to mammalian collagens in tissue engineering. The aim of the present study was to develop a biphasic scaffold from exclusively marine collagens supporting both osteogenic and chondrogenic differentiation and to find a suitable setup for in vitro chondrogenic and osteogenic differentiation of human mesenchymal stroma cells (hMSC). Methods: Biphasic scaffolds from biomimetically mineralized salmon collagen and fibrillized jellyfish collagen were fabricated by joint freeze-drying and crosslinking. Different experiments were performed to analyze the influence of cell density and TGF-β on osteogenic differentiation of the cells in the scaffolds. Gene expression analysis and analysis of cartilage extracellular matrix components were performed and activity of alkaline phosphatase was determined. Furthermore, histological sections of differentiated cells in the biphasic scaffolds were analyzed. Results: Stable biphasic scaffolds from two different marine collagens were prepared. An in vitro setup for osteochondral differentiation was developed involving (1) different seeding densities in the phases; (2) additional application of alginate hydrogel in the chondral part; (3) pre-differentiation and sequential seeding of the scaffolds and (4) osteochondral medium. Spatially separated osteogenic and chondrogenic differentiation of hMSC was achieved in this setup, while osteochondral medium in combination with the biphasic scaffolds alone was not sufficient to reach this ambition. Conclusions: Biphasic, but monolithic scaffolds from exclusively marine collagens are suitable for the development of osteochondral constructs.

## 1. Introduction

Collagen is one of the most frequently applied biomaterials for biomedical research as well as clinical applications [1,2]. The main industrial sources of collagen are bovine and porcine tissues; however, there is increasing demand for alternative sources. Marine collagens which can be obtained from both invertebrates and vertebrates [3,4] show promising features and have the potential to overrule mammalian collagens in biomedical applications for several reasons. Marine collagens do not bear the risk of disease translation and are not allergy-causing; they are not subjected to ethical or religious concerns, show low inflammatory response and can be obtained with high yield [1]. From the beginning of the 21st century research on marine collagens has continuously emerged [5,6]. Scaffolds for tissue engineering applications are increasingly developed from collagens of marine origin, such as from fish collagen, collagen of marine sponges, jellyfish collagen and collagen from marine gastropods [5]. In two own studies we applied marine collagens for the fabrication of porous scaffolds: first we adapted the procedure of biomimetic mineralization of bovine collagen to collagen of the Atlantic salmon *Salmo salar* and prepared mineralized porous scaffolds from salmon collagen for the application in bone tissue engineering [7]. A second study applied collagen of the jellyfish *Rhopilema esculentum* which is structurally similar to human collagen II [8] for the preparation of porous scaffolds to be used for chondral tissue engineering [9]. In the present study, we combined fibrillized jellyfish collagen with biomimetically mineralized salmon collagen to a biphasic scaffold suitable for osteochondral defect regeneration. The applied technique was already described in 2007 for the generation of biphasic, but monolithic scaffolds from mineralized bovine tendon collagen and fibrillized collagen from calf skin/hyaluronic acid composite [10]. Joint freeze-drying and chemical crosslinking of the two different phases resulted in a scaffold material which overcame the risk of delamination of the mineralized and non-mineralized phases, since the scaffolds consisted of a unified whole [10]. Challenge in the fabrication of biphasic scaffolds for the regeneration of both cartilage and the subchondral bone layer is the mechanically stable conjunction of the different phases, which have different mechanical properties to mimic the chemical nature of elastic, water-rich chondral ECM, and the stiff, mineralized bone ECM. The main challenge, however, is the simultaneous chondrogenic and osteogenic differentiation, guided by the scaffold properties which should recapitulate the native milieu of bone and cartilage development [11,12,13,14]. Furthermore, when osteochondral tissue engineering constructs are differentiated in vitro, a suitable osteochondral medium should be developed [15,16]. The aim of the present study was to generate biphasic, but monolithic porous scaffolds from fibrillized jellyfish collagen for the chondral part and biomimetically mineralized salmon collagen for the bony part. Multipotent human mesenchymal stromal cells (hMSC) were differentiated simultaneously into chondrocytes and osteoblasts, respectively, in this biphasic scaffold in vitro. To generate a microenvironment, which guides the cells to the two differentiation lineages, we inserted the cells into the chondral part with a tenfold higher cell density compared to the bony part and, furthermore, used an alginate hydrogel for embedding the cells in the porous chondral part. Furthermore, an osteochondral medium was developed and sequential seeding of the scaffold phases with pre-differentiated MSC was performed.

## 2. Results

### 2.1. Preparation and Characterization of Biphasic Scaffolds from Marine Collagen

**Figure 1:**
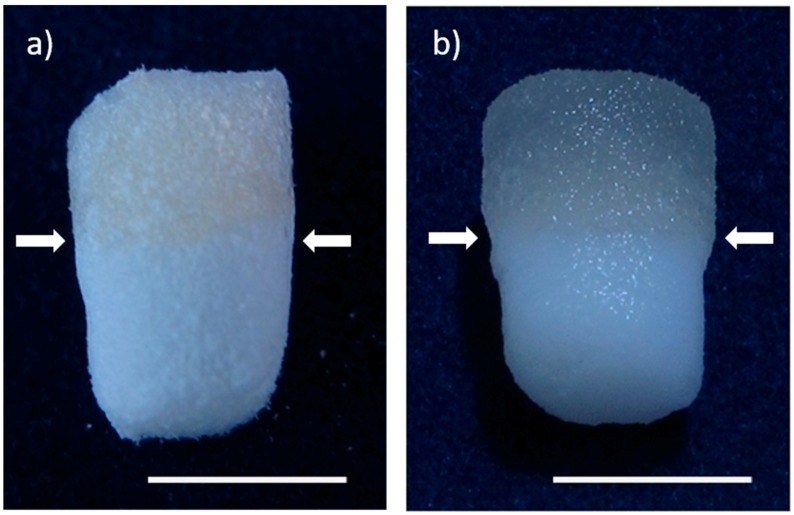
Biphasic scaffolds from mineralized salmon collagen (**lower layer**) and fibrillized jellyfish collagen (**upper layer**) in dry state (**a**) as well in wet state (**b**). Arrows indicate the transition zone between the layers. Scale bar represents 5 mm.

**Figure 2:**
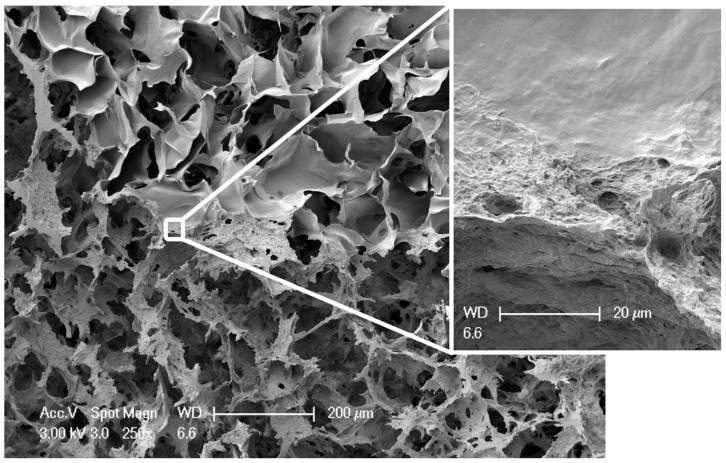
SEM image of a cross section of a biphasic scaffold at the transition zone between jellyfish collagen (**upper layer**) and mineralized salmon collagen (**lower layer**). Scale bar represents 200 µm. Insert showing the transition zone with higher magnification; scale bar represents here 20 µm.

Biphasic, but monolithic scaffolds, composed of biomimetically mineralized salmon collagen [7] and fibrillized jellyfish collagen [9] were successfully obtained by overlaying the two different phases as liquid suspensions, joint freeze-drying and crosslinking. Figure 1 shows the morphology of these scaffolds, both in dry and wet state. Although the stronger swelling of the jellyfish collagen phase is clearly visible, the stability of the whole scaffold is not affected by the different swelling behavior of the phases. This becomes especially obvious, when the microstructure of the scaffolds is analyzed. Interconnecting pores were verified in the transition zone between the two phases (Figure 2). Scanning electron microscopy (SEM) images show the smooth surface of the non-mineralized jellyfish collagen pore walls, as well as the rough morphology of the mineralized salmon collagen, which originates in the presence of hydroxyapatite nanocrystals, decorating the surface (Figure 2).

### 2.2. Evaluation of Optimal Seeding Density for the Osteogenic Differentiation of hMSC

**Figure 3:**
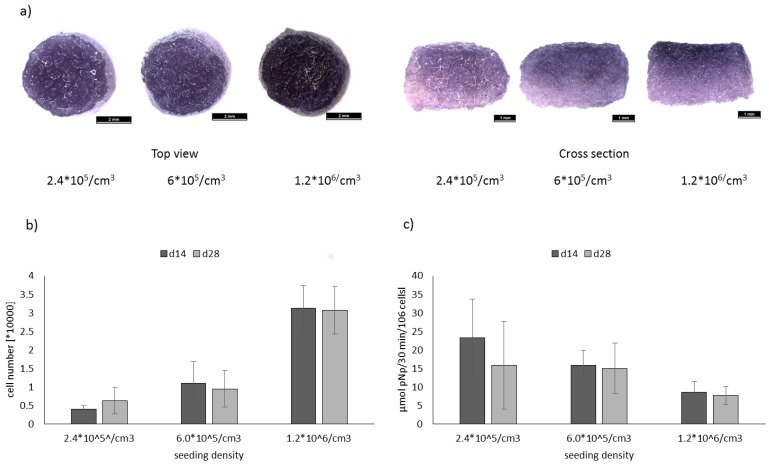
(**a**) MTT staining of osteogenically differentiated hMSC seeded in mineralized salmon collagen scaffolds at different densities and cultivated for 28 days; (**b**) cell number, calculated from DNA content and (**c**) specific ALP activity (ALP activity related to cell number) of osteogenically differentiated hMSC in salmon collagen scaffolds with different seeding densities after 14 and 28 days of cultivation, *n* = 3, mean +/− standard deviation. Significances from 2-way ANOVA were as follows: Cell number: d14 ↔ d28 n.s.; 2.4 × 10^5^ ↔ 6 × 10^5^ n.s., 2.4 × 10^5^ ↔ 1.2 × 10^6^ *p* < 0.05; 6 × 10^5^ ↔ 1.2 × 10^6^ *p* < 0.05, specific ALP activity: d14 ↔ d28 n.s.; 2.4 × * 10^5^ ↔ 6 × * 10^5^ n.s., 2.4 × 10^5^ ↔ 1.2 × 10^6^ *p* < 0.05; 6 × 10^5^ ↔ 1.2 × 10^6^ n.s.

Single scaffolds from mineralized salmon collagen were seeded with hMSC at three different densities, 2.4 × 10^5^ cells/cm^3^, 6 × 10^5^ cells/cm^3,^ and 1.2 × 10^6^ cells/cm^3^ and cultivated under osteogenic stimulation for 14 and 28 days. Number of viable cells, visualized by MTT staining (Figure 3a) was still highest in the scaffolds with the highest initial seeding density after 28 days of osteogenic stimulation. Quantitative analysis of cell number (Figure 3b) confirmed these findings. Furthermore, it was shown, that cell number did not increase between d14 and d28 of cultivation. Osteogenic differentiation was evaluated by quantification of specific alkaline phosphatase (ALP) activity (Figure 3c). Highest specific ALP activities were obtained for the constructs with the lowest seeding density; however, due to high variations between single samples the effect was not statistically significant. Furthermore, the ALP activity in scaffolds seeded with the lowest cell density was close to the detection limit of the colorimetric test. Therefore, for the seeding of biphasic scaffolds, the intermediate cell density (6 × 10^5^ cells/cm^3^) was chosen, since specific ALP activity was still higher compared to the highest seeding density.

### 2.3. Influence of TGF-β on Osteogenic Differentiation

**Figure 4:**
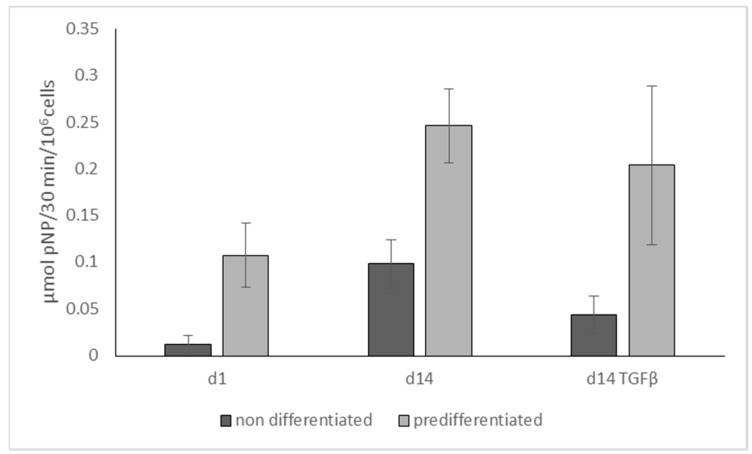
Specific ALP activity of hMSC which were cultivated in monophasic scaffolds from mineralized salmon collagen under osteogenic stimulation (10^−7^ M Dex, 10 mM β-GP, 12.5 µg/mL AAP = osteogenic supplements, OS+) in the presence and absence of 10 ng/mL TGF-β3. Dark bars: cells were prestimulated with OS+ in monolayer culture for 9 days before seeding the scaffolds. Cells of two different donors were used, each *n* = 3 per condition. ALP activity was related to cell number. Values are presented as mean (*n* = 6) +/− standard deviation. Significances from 2-way ANOVA were as follows: d1 ↔ d14 *p* < 0.001; d1 ↔ d14TGFβ *p* < 0.05; d14 ↔ d14TGFβ *p* < 0.05; non-differentiated ↔ pre-differentiated *p* < 0.001.

We tested the influence of TGF-β3 on the osteogenic differentiation of hMSC, seeded in monophasic scaffolds from mineralized salmon collagen. Specific ALP activity after 14 days of osteogenic stimulation showed a significant (*p* < 0.05) decrease in the presence of TGF-β3 both for hMSC and osteogenically prestimulated hMSC (Figure 4). The experiment was also performed to test the effectivity of osteogenic pre-stimulation in the monolayer, and a significantly (*p* < 0.001) higher specific ALP activity was detected both at day 1 and day 14.

### 2.4. Chondrogenic and Osteogenic Differentiation of hMSC in Biphasic Marine Scaffolds

**Figure 5:**
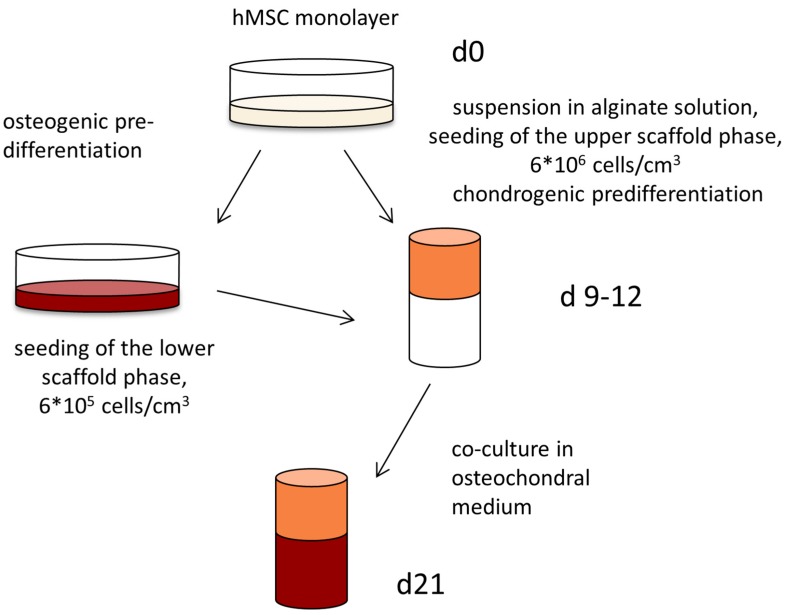
Pre-differentiation and sequential seeding of hMSC onto biphasic scaffolds from jellyfish collagen and mineralized salmon collagen.

A sequential seeding procedure for the biphasic collagen scaffolds was performed (Figure 5). In the first step of sequential cultivation, hMSC were suspended in alginate solution, the jellyfish collagen phase of the biphasic scaffolds was infiltrated with this mixture with a cell density of 6 × 10^6^ cells/cm^3^ and the constructs were cultivated with complete chondrogenic medium for 9 to 12 days. At the same time, hMSC from the same batch were seeded into flasks and cultivated in the presence of osteogenic medium for 9 to 12 days. After 9 to 12 days of pre-stimulation, the osteogenically induced cells were seeded into the mineralized salmon collagen layer of the biphasic scaffolds with an initial cell density of 6 × 10^5^ cells/cm^3^. The constructs were cultivated until d21 from the initial seeding with osteochondral medium, containing 5 ng/mL TGF-β3, ITS, 10^−7^ M Dex and 50 µg/mL AAP. 

**Figure 6:**
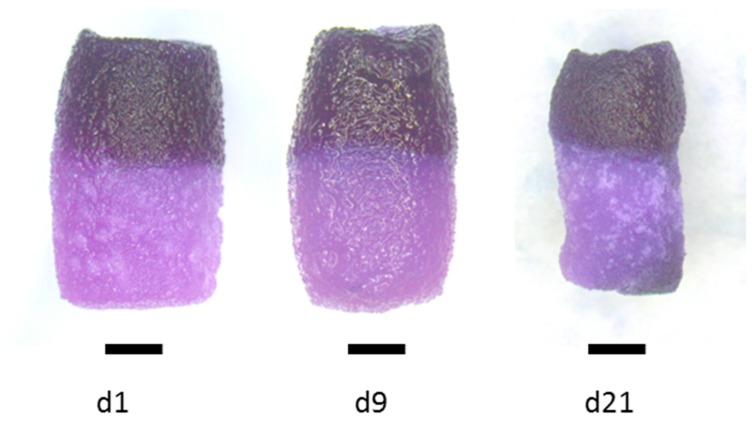
MTT Staining of viable cells in biphasic collagen scaffolds, freshly seeded with alginate-embedded hMSC (d1), after 9 days of chondrogenic differentiation (d9) and after 21 days of cultivation, seeded with osteogenically pre-differentiated hMSC at day 9. Scale bars represent 2 mm.

**Figure 7:**
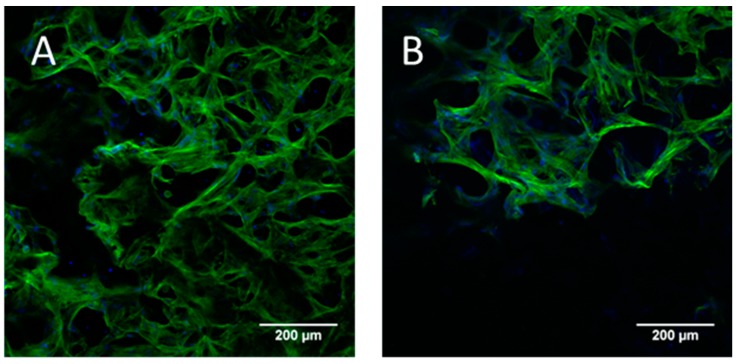
(**A**) cLSM images of a cross section of biphasic collagen scaffold seeded with hMSC after 9 days of chondrogenic differentiation; (**B**) transition area between jellyfish collagen (upper) and salmon collagen (lower) phase, after 9 days of chondrogenic differentiation before seeding of osteogenic cells, cytoskeleton stained with Alexa Fluor 488 phalloidin (green), nuclei stained with DAPI (blue). Scale bars represent 200 µm.

The biphasic constructs were stable during the whole cultivation period. MTT staining of viable cells after 1 and 9 days of cultivation demonstrated that the chondrogenically induced cells did not migrate out of the jellyfish collagen/alginate phase to the mineralized salmon collagen phase below (Figure 6). This was confirmed by confocal laser scanning microscopic (cSM) investigations at the area between the two phases (Figure 7).

**Figure 8:**
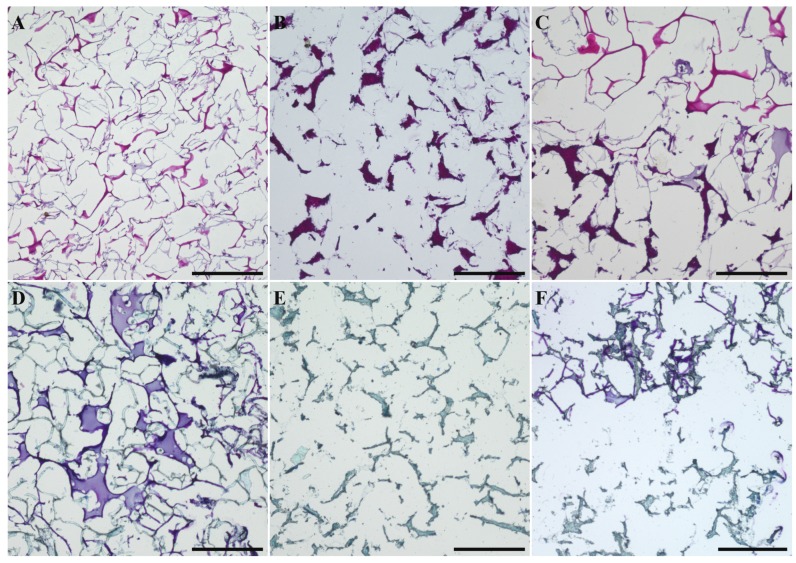
Histological sections of biphasic scaffolds after 21 days of osteochondral cultivation: Haematoxylin/Eosin staining of (**A**) jellyfish collagen phase, (**B**) mineralized salmon collagen phase and (**C**) transition zone between jellyfish collagen part (**top**) and mineralized salmon collagen part (**bottom**). Deeply purple stained regions in (**B**,**C**) represent mineralized salmon collagen which is stained by haematoxylin too. Toluidine blue staining of (**D**) jellyfish collagen phase, (**E**) mineralized salmon collagen phase and (**F**) transition zone between jellyfish collagen part (**top**) and mineralized salmon collagen part (**bottom**).

At the end of the cultivation, a contraction of the biphasic scaffolds was visible. This contraction has been observed before in monophasic collagen scaffolds and it has been shown to be diminished with the application of alginate as cell carrier. Histological sections of biphasic scaffolds after 21 days of cultivation showed the presence of cells in both phases (Figure 8A–C). Furthermore, toluidine blue staining of histological sections revealed the production of cartilage extracellular matrix in the chondrogenic part of the scaffold (Figure 8D–F). The cellularity of the constructs is considerably lower compared to pellet cultures of chondrogenically stimulated cells. However, we have shown in a previous study, that higher cell densities in porous collagen scaffolds did not increase extracellular matrix production per cell [9].

**Figure 9:**
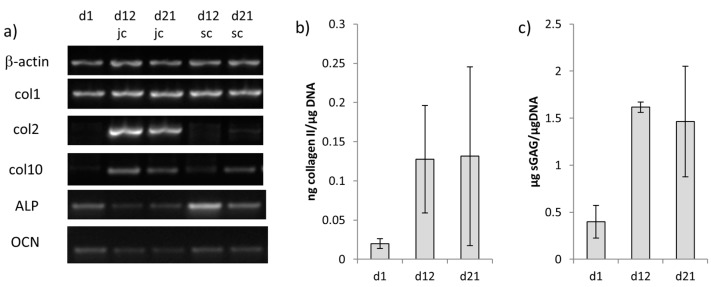
(**a**) RT-PCR products of different osteogenic and chondrogenic marker genes jc = jellyfish collagen, sc = mineralized salmon collagen; (**b**) Collagen II content in the jellyfish collagen phase of biphasic scaffolds, detected by ELISA; (**c**) concentration of sulfated glycosaminoglycanes content in the jellyfish collagen phase of biphasic scaffolds. *n* = 3, mean +/− standard deviation.

Chondrogenic differentiation of the cells in the jellyfish collagen/alginate phase was verified by gene expression analysis of collagen II, which was also detected at protein level (Figure 9).

Gene expression of collagen II increased from d1 to d12, but decreased slightly during the cultivation of the biphasic constructs in the osteochondral medium. Similar results were obtained for the quantification of collagen II by ELISA; however, there were always high variations between the different samples (Figure 9b). Additionally, the production of sulfated glycosaminoglycans increased during chondrogenic differentiation of jellyfish collagen/alginate embedded hMSC, but it did not further increase during cultivation in the osteochondral medium (Figure 9c).

Osteogenic differentiation of hMSC in the mineralized salmon collagen phase was demonstrated by ALP gene expression, which was strongest immediately after seeding of the biphasic scaffolds with osteogenically pre-differentiated hMSC. During further cultivation in osteochondral medium the ALP gene expression decreased (Figure 9a). Furthermore, gene expression of osteocalcin was analyzed in both scaffold parts, which was relatively low in all examined samples, however, cells in the chondrogenic layer showed down-regulation of osteocalcin (Figure 9).

## 3. Discussion

Biphasic, but monolithic scaffolds, exclusively from the marine biopolymers jellyfish collagen, biomimetically mineralized salmon collagen and alginate were fabricated for the first time. Due to the concerted freeze drying of the two phases before crosslinking the layers were tightly connected showing interconnecting pores through the area of the different layers. In the wet state, the two layers showed different swelling behavior. While the biomimetically mineralized salmon collagen phase did not swell, the volume of the fibrillized jellyfish collagen phase increased somewhat in wet state. The reason for the different swelling behavior could be the difference between the collagen types. Collagen II was shown to have higher swelling capacity compared to collagen I [17]. Furthermore, the presence of nanocrystalline hydroxyapatite connected to the collagen fibers of the salmon collagen could be responsible to the reduced swelling in this phase. Nevertheless, the different swelling behavior did not impair the union between the two phases, which have been cross-linked with higher EDC concentrations compared to the monophasic scaffolds. Despite the high concentration of carbodiimide for crosslinking to allow the tight union between the phases, we did not detect any negative effects on cytocompatibility. In a similar approach to our method mineralized and non-mineralized collagen (from equine origin) were combined to a triphasic scaffold for osteochondral regeneration [18]. In contrast to our study, crosslinking of the single layers was performed before freeze-drying, which required an additional knitting procedure to anchor the layers. Biphasic scaffolds from collagen I in the chondrogenic part and collagen I mineralized with Mg^2+^ substituted hydroxyapatite in the osteogenic part were recently prepared by Sartori and co-workers [19]. Also in this study, the collagen layers were cross-linked before freeze-drying. Nevertheless, the resulting biphasic scaffolds were stable enough to withstand subcutaneous implantation in mice for up to 8 weeks. In vitro investigations for MSC differentiation, however, were only performed with monophasic scaffolds in this study.

For the generation of osteochondral tissue constructs in vitro progenitor cells like human mesenchymal stroma cells need to be differentiated into two different lineages in spatially separated phases of the same scaffold. Optimally, scaffold structure and chemical composition provide the necessary stimuli for differentiation into the osteogenic and chondrogenic cell line. Jellyfish collagen from R. esculentum has a similar structure to human collagen II, since it consists of α-chain homotrimers and shows a degree of glycosylation similar to that of vertebrate collagen type II [20]. Chondrogenic differentiation is triggered by clustering of hMSC, which can be realized by 3D pellet formation or seeding of scaffolds with high cell densities [21]. Additionally, embedding of cells into alginate hydrogel, infiltrated into porous collagen scaffolds, induced a chondrogenic phenotype and increased collagen II expression of the cells [22]. Therefore, the upper (chondral) phase of the marine biphasic scaffolds was seeded with hMSC in high density (6 × 10^6^ cells/mL) which were incorporated into alginate hydrogel completely filling the pores of the jellyfish collagen part of the scaffolds. Osteogenic differentiation is favored by the presence of hydroxyapatite, and it has been shown in porous collagen scaffolds in vitro, that specific ALP activity which is the main osteogenic marker increases with decreasing seeding density [23]. Similar results were also obtained in the present study for monophasic scaffolds from mineralized salmon collagen (Figure 3c). The lower phase of the biphasic marine scaffolds was therefore seeded with hMSC in a tenfold lower density compared to the chondral part, and the cells were allowed to attach directly at the pore walls of the mineralized salmon collagen without addition of alginate. Nevertheless, as shown in our previous studies with monophasic scaffolds from jellyfish collagen, scaffold structure and composition as well as cell density alone are not sufficient to induce chondrogenic differentiation of hMSC. Likewise, biomimetically mineralized collagen scaffolds, despite their bone-like composition of collagen and nanocrystalline hydroxyapatite, are not osteoinductive. The addition of osteogenic stimuli is necessary to induce osteogenic differentiation of MSC seeded in the scaffolds. For simultaneous osteogenic and chondrogenic differentiation of hMSC in the marine biphasic scaffolds in vitro, a suitable osteochondral medium needed to be developed. Cell culture media for the chondrogenic and osteogenic differentiation of MSC have equal and distinct components [24]. While dexamethasone and ascorbate are included in both chondrogenic and osteogenic differentiation medium, both media have exclusive components (Table 1).

To develop an osteochondral medium supporting both osteogenic and chondrogenic differentiation of MSC (additionally to the stimuli, which are exerted by the scaffold matrix) it is not sufficient just to combine the single media 1:1. In a previous study we have analyzed the impact of FCS on the chondrogenic differentiation of hMSC in monophasic scaffolds of jellyfish collagen [25] and demonstrated, that even small amounts of FCS (2%) in the culture medium significantly decrease the mRNA expression of chondrogenic markers. In addition, reduction of glucose content caused a decreased mRNA expression of chondrogenic markers as well as a decreased extracellular matrix production of the chondrogenically differentiated cells. In contrast, osteogenic differentiation of hMSC in mineralized salmon collagen scaffolds was not affected when the glucose concentration of the medium was increased (data not shown). Furthermore, osteogenic differentiation of hMSC is even favored by low serum conditions (1% and 5% compared to 10%) [26]. Β-glycerophosphate which is an integral component of osteogenic differentiation media to provide a phosphate source for the mineralizing osteoblasts, is regarded as hypertrophy promoting reagent for chondrocytes [27]. The main component of chondrogenic differentiation medium, TGF-β, was shown to cause a downregulation of ALP expression [28], which was also found in our study for MSC seeded in scaffolds from mineralized salmon collagen (Figure 4). Based on these results we propose an osteochondral medium containing a reduced amount of TGF-β (5 ng/mL), no FCS, high glucose content, bovine serum albumin, as well as the factors stimulating both osteogenic and chondrogenic differentiation: dexamethasone and ascorbic acid-2-phosphate. However, even the combination of (1) different seeding densities in the osteogenic and chondrogenic part (2) different scaffold layer composition (3) alginate infiltration in the chondrogenic part preventing cell adhesion to the scaffolds pores and therefore supporting chondrogenic cell phenotype and (4) osteochondral medium was not sufficient to stimulate osteogenic and chondrogenic differentiation spatially separated in the respective scaffold parts. Similar results were obtained by Gupta et al. who tried to simultaneously differentiate rat MSC into chondrogenic and osteogenic lineage in gradient PLGA scaffolds with encapsulated chondroitin sulfate for chondrogenic priming as well as tricalcium phosphate for osteogenic priming [29]. The authors admittedly reported a better interaction of cells with the materials and a greater cellularity, but it was not possible to drive differentiation specifically into either of the planned directions. Caliari and Harley investigated the impact of scaffold local biophysical properties like mineral content and density on hMSC differentiation in the presence of mixed soluble signals for osteogenic and chondrogenic differentiation [30]. Unexpectedly, the authors observed an increased osteogenic response just in nonmineralized scaffolds with low density, which were intended to induce chondrogenic differentiation. Therefore, in the present study, a sequential seeding and pre-differentiation approach was developed. It has been shown before, that temporal stimulation of chondrogenic cells with TGF-β is sufficient to induce the chondrogenic phenotype in MSC. Buxton and co-workers stimulated hMSC in hydrogels with TGF-β1 and demonstrated, that total production of collagen II after three weeks of cultivation was not decreased in comparison to the controls, when TGF-β was withdrawn after 7 days of prestimulation [31]. Likewise Fensky and co-workers demonstrated, that a 10 day stimulation of hMSC embedded in a collagen I hydrogel is sufficient to induce upregulation of the chondrogenic marker genes for collagen II and aggrecan after three weeks of cultivation [32]. Short delivery of high TGF-β doses (100 ng/mL) for 7 days on bovine MSC embedded in hyaluronic acid hydrogels was sufficient to induce and maintain the chondrogenic phenotype over a period of 9 weeks [33]. Chondrogenically pre-differentiated hMSC maintained their chondrogenic potential after embedding in methacrylated hyaluronic acid gels [34]. Osteogenic pre-differentiation of MSC before application in bone regeneration has successfully been applied by several groups. Peters et al. demonstrated that the injection of osteogenically pre-differentiated MSC enhanced healing of a critical bone defect in rats [35]. It is hypothesized that the application of osteogenically pre-differentiated instead of undifferentiated hMSC may prevent the transplanted cells from neoplasia and tumor formation [36]. Osteogenic pre-differentiation of hMSC has been successfully applied in our study. Osteogenically pre-differentiated hMSC seeded into monophasic scaffolds from mineralized salmon collagen showed significantly increased ALP activity both at the start of the 3D cultivation and after 14 days. Furthermore, the negative effect of TGF-β on ALP activity is less pronounced for osteogenically pre-differentiated cells compared to non-differentiated MSC, which is a further point to apply osteogenic pre-differentiation for osteochondral constructs. Osteocalcin gene expression, which is a further marker of osteogenic differentiation, was quite low in the biphasic scaffolds. This is in accordance to a fundamental study of Jaiswal and co-workers, demonstrating that vitamin D3 is necessary to induce adequate osteocalcin expression in osteogenically differentiated hMSC in vitro [37]. We refrained from adding vitamin D3 to the osteochondral medium to preserve the chondrogenic phenotype in the chondral layer. However, it might be beneficial to include vitamin D3 into the osteogenic pre-differentiation medium in future. In the present study, osteogenic and chondrogenic pre-differentiation was performed simultaneously. While chondrogenic pre-differentiation was realized already during cultivation of alginate-embedded hMSC in the jellyfish collagen part of the biphasic scaffold, osteogenic pre-differentiation of hMSC of the same batch was performed in the monolayer. Gene expression analysis revealed collagen II expression exclusively in the chondral part of the scaffolds; however, there was a slight decrease in collagen II expression during the cultivation with osteochondral medium in the presence of osteogenically pre-differentiated cells. Likewise, collagen II production on protein level and production of sGAG were somewhat decreased during co-culture (Figure 9). It has been demonstrated that continuous treatment of hMSC pellet cultures with TGF-β provided significantly higher production of extracellular matrix and expression of chondrogenic genes compared to short-time TGF-β supplementation of 3 and 10 days [38]. Possibly, the reduced TGF-β supplementation during osteochondral cultivation was not sufficient to stabilize the chondrogenic phenotype at the starting level. Similar observations were made for the osteogenic part. Highest ALP expression was detected immediately after seeding of the biphasic scaffolds, followed by a decrease of ALP expression during further osteochondral stimulation. Since ALP is an early osteogenic marker, the decrease could also relate to further differentiation along the osteogenic lineage. However, it has to be noted, that the applied osteochondral medium does not provide optimal conditions for osteogenic differentiation. Nevertheless, the RNA amount isolated from the mineral part of the biphasic scaffolds after 3 weeks of cultivation was not reduced compared to the start of 3D cultivation, suggesting that the osteogenically pre-differentiated cells survived the osteochondral conditions with serum deprivation. After three weeks of cultivation osteochondral constructs were obtained in vitro with spatial separated expression of chondrogenic ECM and osteogenic differentiation.

## 4. Materials and Methods 

### 4.1. Preparation of Biphasic Scaffolds

Biphasic but monolithic collagen scaffolds were prepared by overlaying suspensions of biomimetically mineralized salmon collagen and fibrillized jellyfish collagen in the cavities of a 96-well plate. Biomimetically mineralized collagen and fibrillized jellyfish collagen suspensions were prepared as already published [7,9]. After freezing of the overlayed samples at 1 K/min to a final temperature of −20 °C lyophilization was conducted for about 24 h (Alpha 1–2, Christ). For stabilization the scaffolds were cross-linked with a 30 g/L solution of *N*-(3-dimethylaminopropyl)-*N*′-ethylcarbodiimide (EDC) hydrochloride in 80% *v*/*v* ethanol for 12 h. Subsequently, the scaffolds were rinsed thoroughly in deionized water, 1% glycine solution, once again in water and finally freeze-dried.

### 4.2. Cultivation of hMSC

Bone marrow derived hMSC harvested from the iliac crest of two healthy donors were kindly provided by the group of Prof. Martin Bornhäuser (Medical Clinic I, University Hospital Dresden). Written informed consent from the donors was obtained for the use of these samples in research. Cells were characterized as hMSC according to the criteria of the International Society of Cellular Therapy [39]. All procedures were approved by the Ethical Commission of the Medical Faculty of Technische Universität Dresden. Cells were expanded in Dulbecco′s modified Eagle′s Medium (DMEM, Gibco) supplemented with 10% fetal calf serum, 2 mM L-glutamine, 100 U/mL penicillin and 100 µg/mL streptomycin (all from Biochrom, Berlin, Germany) (expansion medium) until passage five. 

General seeding preparation for porous collagen scaffolds: Scaffolds were immersed with expansion medium for 24 h, which was removed before seeding wet scaffolds were placed on sterile filter paper to remove excess immersion medium from the scaffold pores.

### 4.3. Osteogenic Differentiation of hMSC in Mineralized Salmon Collagen Scaffolds

Monophasic scaffolds from mineralized salmon collagen (d = 6 mm, h = 3 mm) were prepared as already published [7] and sterilized by γ-irradiation before use in cell culture. Scaffolds were seeded with 2 × 10^4^, 5 × 10^4,^ and 1 × 10^5^ cells in 50 µL of expansion medium. After 30 min of initial adhesion, further 500 µL of expansion medium were added to each scaffold. Cell-seeded scaffolds were cultivated for up to 4 weeks, with medium change every 3–4 days. Osteogenic differentiation medium contained Minimal essential medium α-modification (α-MEM) (Biochrom Berlin, Germany), supplemented with 10% fetal calf serum, 2 mM L-glutamine, 100 U/mL penicillin and 100 µg/mL streptomycin (all from Biochrom), 10^−7^ M Dex, 12.5 µg/mL AAP and 10 mM β-GP (all from Sigma, Taufkirchen, Germany). Since we wanted to analyze the effect of TGF-β on the osteogenic differentiation additionally 10 ng/mL TGF-β3 (Miltenyi, Bergisch Gladbach, Germany) were added to one experimental group. After 1, 14 and 28 days samples for DNA and ALP quantification were washed twice and frozen at −80 °C.

### 4.4. Cultivation of Osteochondral Constructs

Biphasic scaffolds from jellyfish collagen and mineralized salmon collagen (d = 6 mm, h = 8 mm) were sterilized by γ-irradiation before use in cell culture. Sodium alginate (Sigma) was dissolved in Ca^2+^ free DMEM (Sigma) without any further supplements at 12 mg/mL and the solution was filtered through a syringe filter with 0.45 µm pore size. 5 × 10^5^ cells were suspended in 50 µL of alginate solution and the suspension was applied to the top of each biphasic scaffold. With 6 mm diameter and 3 mm height the chondral phase of the biphasic scaffold has an approximate volume of 85 mm^3^ resulting in a seeding density of 6 × 10^6^ cells/cm^3^. After 15 min of incubation at 37 °C, 1 mL of sterile CaCl_2_ (100 mM) solution was added and the constructs were incubated for further 15 min for the formation of alginate gel. The constructs were washed with expansion medium and cultivated further with chondrogenic differentiation medium (DMEM high glucose (Gibco, Dublin, Ireland, distributed by Thermo Fisher, Waltham, MA, USA), 100 U/mL penicillin, 100 µg/mL streptomycin, 1% ITS-X-Mix (Gibco; results in concentrations of 10 µg/mL insulin, 5.5 µg/mL transferrin, 6.7 ng/mL sodium selenite and 2 µg/mL ethanolamine in the medium), 0.35 µM proline, 50 µg/mL AAP, 10^−7^ M Dex, 0.15% bovine serum albumin (BSA) (all Sigma) and 10 ng/mL TGF-β3 (Miltenyi)) for 9–12 days with medium changes every 3–4 days. At the same time, hMSC from the same batch were cultivated in T-flasks with α-MEM supplemented with 10% fetal calf serum, 2 mM L-glutamine, 100 U/mL penicillin and 100 µg/mL streptomycin, 10^−7^ M Dex, 12.5 µg/mL AAP and 10 mM βGP. After 9 to 12 days of separate cultivation, biphasic scaffolds seeded with chondrogenically pre-differentiated hMSC were gently dried on sterile filter paper, flipped, that the mineralized layer was on top of the scaffold and were seeded in the mineralized salmon collagen layer with 5 × 10^4^ osteogenically pre-differentiated hMSC in 50 µL cell culture medium, resulting in a seeding density of 6 × 10^5^ cells/cm^3^. The constructs were cultivated up to 21 days of total cultivation time with osteochondral medium consisting of DMEM high glucose supplemented with 100 U/mL penicillin, 100 µg/mL streptomycin, 1% ITS-X-Mix, 5 ng/mL TGF-β3, 10^−7^ M Dex, 0.35 µM proline, 0.15% BSA and 50 µg/mL AAP. For each time point of the experiment 9 biphasic scaffolds were used (3 for gene expression analysis, 3 for measurement of collagenII and sGAG, 1 for MTT staining, 1 for fluorescence staining and 1 for histological staining).

### 4.5. MTT Staining

To detect the distribution and amount of viable cells, medium of cell-seeded monophasic and biphasic scaffolds was supplemented with 1.2 mM 3-(4,5-dimethylthiazol-2-yl)-2,5-diphenyltetrazolium bromide (MTT; Sigma), followed by further incubation at 37 °C for 4 h. Cell seeded scaffolds were imaged using a Leica stereomicroscope.

### 4.6. Fluorescence Staining and Confocal Laser Scanning Microscopy

Cell-seeded scaffolds were fixed with 4% buffered formaldehyde and permeabilized with 0.2% Triton X-100 (Sigma) in Hank’s balanced salt solution (HBS) (Gibco). Autofluorescence was blocked with, 30 min incubation in 3% BSA in HBS. Staining of cytoskeleton was performed with Alexa Fluor 488 phalloidin and staining of the nuclei with 0.3 µM DAPI (4′,6-diamidino-2-phenylindole dihydrochloride; both Invitrogen) in HBS. Samples were imaged using a confocal laser scanning microscope LSM 510 (Zeiss, Jena, Germany) applying an excitation/emission wavelength of 405/461 nm (diode laser) for DAPI and 488/519 nm (argon laser) for Alexa Fluor 488.

### 4.7. Gene Expression Analysis

Biphasic scaffolds were divided with a scalpel into the two phases prior to RNA extraction, for which three samples of each experimental group were used. During the RNA isolation procedure, cell lysates of the three samples of each group were pooled.

To dissolve the alginate in alginate-containing jellyfish collagen constructs, samples were mixed thoroughly with 55 mM sodium citrate (Fluka, distributed by Sigma-Aldrich, Taufenkirchen, Germany) with 0.9% sodium chloride (Sigma), in DEPC-water (Gibco), incubated at 37 °C for 45 min and afterwards mixed thoroughly again. Cells in the supernatant were centrifuged at 3600 rpm for 10 min, resuspended in PBS, and centrifuged again. Both the pellet and the collagen scaffold were treated with lysis buffer from peqGOLD MicroSpin total RNA Kit (Peqlab, Erlangen, Germany). Lysates from the pellets and scaffolds were pooled and RNA was extracted according to the manufacturer′s instructions of the kit. To isolate RNA from the mineralized salmon collagen phase, cell seeded scaffolds were treated with lysis buffer from the RNA kit directly.

For polymerase chain reaction (PCR) 200 ng per experimental condition were transcribed into cDNA in a 20 μL reaction mixture containing 200 U of superscript II reverse transcriptase, 0.5 mM dNTPs (both Invitrogen), 12.5 ng/μL random hexamers (Eurofins MWG Operon, Ebersberg, Germany) and 40 U of RNase inhibitor RNase OUT (Invitrogen, Carlsbad, CA, USA). 1 µL cDNA in 20 µL reaction mixtures containing specific primer pairs were used for amplification in PCR analysis to detect transcripts of collagen I, collagen IIa, collagen X, ALPL and β-actin, respectively. Primer sequences (Eurofins MWG Operon), annealing temperatures and amplicon sizes for each gene are summarized in Table 2.

For analysis, PCR products were visualized in 2% agarose gels (Ultra PureTMAgarose, Invitrogen).

### 4.8. Analysis of DNA Content, ALP Activity in Monophasic Scaffolds from Mineralized Salmon Collagen sGAG Content and Collagen II Content

Frozen cell-seeded scaffolds were homogenized in ice-cold PBS (2 × 10 s at 5900 rpm) using a Precellys24 apparatus (Peqlab, Erlangen, Germany). After homogenization, 10% Triton X-100 in PBS were added to a final concentration of 1% Triton-X-100 and the samples were incubated on ice for 50 min. ALP activity and DNA content were analyzed from the same lysate. ALP activity was analyzed by conversion of p-nitrophenyl phosphate (Sigma, 1 mg/mL), in 0.1 M diethanolamine pH 9.8, 1% Triton X-100 and 1 mM MgCl_2_, to p-nitrophenol after 30 min of incubation at 37 °C and absorption measurement at 405 nm (Infinite^®^ M200 Pro, Tecan, Männedorf, Switzerland). DNA content was quantified from the same lysate with Quantifluor dye (Promega, Madison, WI, USA) at an excitation/emission wavelength of 485/535 nm. Cell number was calculated from DNA content of defined cell numbers. ALP activity was related to the cell number of the respective sample.

### 4.9. sGAG Content and Collagen II Content in Biphasic Scaffolds

Biphasic scaffolds were divided with a scalpel into the two phases prior to freezing for subsequent biochemical analyzes which included always three biphasic scaffolds per group. After thawing of alginate-containing jellyfish collagen constructs, samples were covered with 500 µL of 55 mM sodium citrate (Fluka) with 0.9% sodium chloride (Sigma), in deionized water, homogenized (1 × 10 s at 5900 rpm, Precellys24, Peqlab) incubated at 37 °C for 30 min and afterwards mixed thoroughly again. After thawing mineralized salmon collagen constructs, samples were covered with 450 µL PBS, homogenized (2 × 10 s at 5900 rpm), 50 µL of 10% Triton X100 were added, and the mixture was incubated for 30 min at 37 °C. All samples were centrifuged at 3600 rpm for 10 min. The supernatants were used for collagen II ELISA, while the pellet was further processed for sGAG determination. Collagen II ELISA was performed as already published [25]. Briefly, each 50 µL of the supernatants was added to wells which were precoated with primary antibody (Mouse Anti-Chick Collagen II Capture Antibody (clone 35; Chondrex, Redmont, WA, USA) diluted 1:500 in PBS). A calibration line was established using human collagen II (Millipore) diluted in PBS with 3% normal goat serum (NGS; Life Technologies, Carlsbad, CA, USA). Afterwards 50 µL of secondary antibody (Biotin-labeled Detection Antibody (Mouse monoclonal anti-Type 2 Collagen (Chondrex)) diluted 1:100 in PBS with 3% NGS) were added. Detection was performed with streptavidin-horseradish-peroxidase (R&D Systems, Minneapolis, MN, USA) and 3,3′,5,5′-tetramethylbenzidin substrate-solution (Sigma). Absorbance was assessed at 450 nm (reference 570 nm) in a microplate reader (Infinite^®^ M200 Pro, Tecan). Absorbance values from scaffolds without cells carried along during the experiments were subtracted as correction factors.

sGAG content from the pellets was quantified as previously described [25]. Briefly, 1 mL of papain digestion solution (containing 125 µg/mL papain, 5 mM EDTA (both from Sigma), 100 mM Na_2_HPO_4_, and 5 mM cystein (both from Carl Roth, Karlsruhe, Germany) in deionized water) were added to each pellet. After incubation at 60 °C for 24 h. 50 µL of the digested solution were subjected to a commercially available sGAG assay (Kamiya, Seattle, WA, USA) according to manufacturer′s instructions. Absorbance was assessed at 610 nm (Infinite^®^ M200 Pro, Tecan).

### 4.10. Histological Investigations on Biphasic Scaffolds

Biphasic scaffolds cultivated for 21 days under osteochondral stimulation were fixed with 4% buffered formaldehyde, dehydrated and embedded in paraffin. 5 µm sections were cut and mounted to cover slides. After deparaffinization, sections were stained with hematoxylin/eosin (H/E) to visualize cell distribution and toluidine blue to visualize the production of cartilage proteoglycans. Stained samples were images using a BZ-9000 (Biorevo) (Keyence, Neu-Isenburg, Germany) microscope.

### 4.11. Statistical Analysis

Statistical analyses for cell number and ALP activity were performed by two-way analysis of variance (ANOVA). Post-hoc analysis was performed in all cases to determine multiple comparisons using the Tukey method (Origin 9.1, OriginLab). Significance levels were set as *p* < 0.05, *p* < 0.01 and *p* < 0.001.

## 5. Conclusions

Biphasic, but monolithic scaffolds exclusively from marine collagens are stable under cell culture conditions for up to three weeks without any delamination of the phases. We have tried to simultaneously differentiate hMSC into osteogenic and chondrogenic lineage spatially separated in the bone and cartilage layer of the marine scaffolds. However, the different chemical nature and mineralization of the layers, as well as different seeding densities, and the application of alginate hydrogel to embed the cells into the jellyfish collagen layer of the scaffold were not sufficient to trigger the differentiation of hMSC adequately into the respective direction. We therefore propose a sequential seeding of the biphasic scaffolds and pre-differentiation of the cells into both osteogenic and chondrogenic lineage to obtain functional osteochondral constructs.

## Figures and Tables

**Table 1:** Main differences in the composition of commonly used osteogenic and chondrogenic differentiation media (FCS = fetal calf serum, ITS = insulin, selen, transferrin mix, AAP = ascorbic acid-2-phosphate, Dex = dexamethasone, β-GP = β-glycerophosphate, TGF-β = transforming growth factor β).

Osteogenic medium	FCS	-	Low glucose	AAP	Dex	β-GP	
Chondrogenic medium	-	ITS	High glucose	AAP	Dex	-	TGF-β

**Table 2:** Primer and conditions for reverse transcriptase PCR.

Marker	Bp	Primer (Forward/Reverse)	Buffer/±Enhancer	T_annealing_	Amplification Cycles
β-Act	234	5′-GGACTTCGAGCAAGAGATGG-3′ 5′-AGCACTGTGTTGGCGTACAG-3′	buffer S/−	55 °C	30x
Col 1	331	5′-GGATGAGGAGACTGGCAAC-3′ 5′-GAAGAAGAAATGGCAAAGAGAAAG-3′	buffer S/−	55 °C	25x
Col 2	388	5′-GAACATCACCTACCACTGCAAG-3′ 5′-GCAGAGTCCTAGAGTGACTGAG-3′	buffer Y/+	60 °C	35x
Col 10	196	5′-GCCCACTACCCAACACCAAGAC-3′ 5′-CCTGGCAACCCTGGCTCTC-3′	buffer S/−	50 °C	30x
ALP	162	5′-ACCATTCCCACGTCTTCACATTTG-3′ 5′-ATTCTCTCGTTCACCGCCCAC-3′	buffer S/−	55 °C	30x
OCN	177	5′-CAA AGG TGC AGC CTT TGT GTC-3′ 5′-TCA CAG TCC GGA TTG AGC TCA-3′	buffer S/−	55 °C	35x
